# Youth perspectives on community health in Nunavik: a community-engaged photovoice project

**DOI:** 10.17269/s41997-022-00687-9

**Published:** 2022-11-08

**Authors:** Madeleine Pawlowski, Mylene Riva, Christopher Fletcher, Marie-Claude Lyonnais, David Arsenault-Hudon

**Affiliations:** 1 Department of Geography, McGill University, Burnside Hall, 805 Sherbrooke Street West, Montreal, QC H3A 0B9 Canada; 2 Institute for Health and Social Policy, McGill University, Burnside Hall, 805 Sherbrooke Street West, Montreal, QC H3A 0B9 Canada; 3 Département de médecine sociale et préventive and Centre de recherche du CHU de Québec – Université Laval, Pavillon Ferdinand-Vandry, Université Laval 1050, avenue de la Médecine, Québec, QC G1V 0A6 Canada; 4 Nunavik Regional Board of Health and Social Services (NRBHSS), P.O. Box 900, Kuujjuaq, QC J0M 1C0 Canada

**Keywords:** Indigenous peoples, Inuit, Adolescent, Qualitative research, Photography, Community-based participatory research, Peuples autochtones, Inuit, adolescents, recherche qualitative, photographie, recherche participative communautaire

## Abstract

**Objective:**

The overall objective of this study was to elicit understandings of community health among Inuit youth aged 12–18 in the region of Nunavik, northern Quebec, through identifying community conditions supporting health from their perspective and exploring how they conceptualize a healthy community.

**Methods:**

In January and February 2020, 51 secondary students from three communities participated in a 1-week participatory photovoice activity during regular class time. Youth participated in three different sessions dedicated to the ethics of taking photographs, taking photos in the community, and group discussions of photographs. Discussions were analyzed via thematic analysis and validated with the youth in the fall of 2020.

**Results:**

Twelve key community conditions supporting health were identified: family, food, culture, language, sense of community belonging, land, housing, services, community, connection, caring and somewhere to go. The youth understood a healthy community to be a place where “nothing was broken” and where community conditions supporting health could be visualized like the rocks in an inuksuk, a stone cairn used by Inuit for wayfinding on the tundra landscape. Participants chose the human form of inuksuk which has become widespread in northern and southern Canadian popular culture.

**Conclusion:**

Findings from this study serve to confirm and strengthen existing models of Inuit health while also raising fresh perspectives and concepts relevant to the younger generation. Images and words of the youth identified in this study may be important in designing effective health promotion strategies that are accessible and relevant to younger generations, thus responding to an important research, programmatic and policy gap.

## Résumé

**Objectif:**

L’objectif général de cette étude était d’expliciter comment des jeunes Inuit âgés de 12 à 18 ans, dans la région du Nunavik au Québec, comprennent la santé en identifiant les conditions des communautés qu’ils estiment favorables à leur santé et en explorant comment ils conceptualisent une communauté en santé.

**Méthodes:**

En janvier et février 2020, 51 élèves du secondaire de trois communautés ont pris part à une activité *photovoice* participative, réalisée sur une période d’une semaine pendant les heures de classe. Durant cette activité, les jeunes ont participé à trois sessions différentes consacrées à l’éthique de la prise de photos, la prise de photos dans la communauté, et des discussions de groupe sur les photos. Une analyse thématique des discussions a été réalisée puis validée avec les jeunes à l’automne 2020.

**Résultats:**

Les jeunes ont identifié douze conditions des communautés qui sont favorables à leur santé : la famille, la nourriture, la culture, la langue, le sentiment d’appartenance à la communauté, le territoire, le logement, les services, la communauté, la connexion, prendre soin, et un endroit où aller. Pour les jeunes, une communauté en santé est un endroit où « rien n’est brisé », où les conditions favorables à la santé peuvent être visualisées comme les pierres d’un inuksuk, un cairn utilisé par les Inuit pour s’orienter dans la toundra. Les participants ont choisi la forme humaine de l’inuksuk qui est répandue dans la culture populaire du nord et du sud du Canada.

**Conclusion:**

Les résultats de cette étude servent à confirmer et à renforcer les modèles existants de la santé des Inuit tout en soulevant de nouvelles perspectives et de nouveaux concepts pertinents pour les jeunes. Les images et les mots des jeunes identifiés dans cette étude peuvent être importants pour la création de stratégies efficaces de promotion de la santé qui sont accessibles et pertinentes pour cette population, répondant ainsi à une importante lacune dans la recherche, les programmes et les politiques.

## Introduction

Despite representing a large and growing segment of the population in Indigenous communities (Statistics Canada, 2017), youth under the age of 18 are often underrepresented in traditional health survey methods including questionnaires and in-depth interviews (Paris & Winn, 2013). To get a complete picture of what makes a community healthy, there is increasing recognition for the need to explore youth perspectives on health and well-being (Ip, 2007; Jennings & Lowe, 2013; Lines et al., 2019; Markus, 2012). Indeed, what is important to the health of adults and elders may not be so for youth, because of their age and the unique historical context in which they live (Wexler, 2009). For example, while culture and language are considered critical components of health to elders, little is known about how these determinants stack up to the world of video games, social media and memes, which uniquely characterize the life of young people. While the words and images youth use to communicate about and understand health can be critical in shaping effective health promotion work (Ramey et al., 2019), these perspectives are rarely explored and documented. Efforts to study these perspectives may be useful in identifying youth-specific determinants and understandings of health to inform community programming.

Along with traditional epidemiological population cohort studies of youth and adults, the *Qanuilirpitaa?* 2017 Nunavik health survey (hereafter Q2017) included a community component meant to provide tools to communities to develop community health programs and promotion activities. The resulting “IQI model” and 8 community determinants of health (Fletcher et al., this issue) drew on existing documents on Inuit health and health determinants (Inuit Tapiriit Kanatami, 2014; Makivik Corporation et al., 2014), consultation with Nunavik communities, and the insights of Inuit culture and language experts. The model offers a multifactorial and dynamic approach to health that is anchored in Inuit culture and language. The present study builds on this culturally and socially informed approach by exploring in more detail with young people how they see and understand community health. As such, it responds to community requests raised over the course of the Q2017 survey for more attention to youth understandings of health.

Youth are more likely to participate in research if the methods are age-appropriate, interesting and engaging (Young et al., 2013). In the field of public health, photovoice has been identified as an effective method for exploring health definitions, priorities and challenges from the perspectives of Indigenous youth (Ip, 2007; Jennings & Lowe, 2013; Lines et al., 2019; Shea et al., 2013; Young et al., 2013). Photovoice is a way of using photography to spark discussion on a topic and a way of showing strengths or opportunities for improvement in a community (Wang & Burris, 1997). By having youth photograph their realities and explore the meanings behind the photos, the technique orients the interpretive process towards the lived realities of participants (Castleden & Garvin, 2008). When thoughtfully adapted to local Indigenous cultural contexts, photovoice may be said to be a culturally safe method (Bennett & Dearden, 2013). Through privileging the voices and vision of Indigenous participants in research, the method can provide counter-discourses to those that predominate in constructing the condition of Indigenous youth, while engaging the storytelling traditions of Indigenous communities (Markus, 2012).

By using a combination of visual, oral and textual approaches, nuanced meanings and complex descriptions of health can emerge, fostering mutual recognition and comprehension within and between communities of people. For example, Gabel and colleagues successfully used photovoice with youth and elders in a southern Labrador Inuit community, drawing out numerous themes that lend themselves to intergenerational health promotion activities (Gabel et al., 2016). In Nunavik, photovoice has been used to understand conceptions of mental health among youth aged 18 to 25 (Perrault Sullivan, 2015).

Capitalizing on the strengths of the photovoice method, the overall objective of this study was to elicit understandings of community health among Inuit youth in Nunavik through (1) identifying community conditions supporting health from their perspective and (2) exploring how they conceptualize a healthy community. Images and words of youth identified in this study may be important in designing effective health promotion strategies that are accessible and relevant to younger generations, thus responding to an important research, programmatic and policy gap.

## Methods

This project was conducted in secondary schools in three communities in Nunavik, using a community-based participatory research (CBPR) (Minkler & Wallerstein, 2011) and strengths-based approach (Mathie & Cunningham, 2003), with a photovoice method (Wang & Burris, 1997). In CBPR, research is developed in a collaborative partnership between researchers and communities, who each bring their unique expertise to the table to work towards answering a question of common interest (Macaulay et al., 1999). Two key community partners were involved in the project: the Nunavik Regional Board of Health and Social Services (NRBHSS) and Kativik Ilisarniliriniq (KI). The NRBHSS is responsible for health and social policy and programming in Nunavik, and KI is the Inuit-run school board. In line with a CBPR approach, the NRBHSS contributed to developing the research methodology, including choosing photovoice as the method for this project. Photovoice is congruent with a CBPR approach because of its ability to effectively balance power and create a sense of ownership over the research process on the part of the participants (Castleden & Garvin, 2008). KI approved the project and helped facilitate relationships with schools. Teachers were integral in helping facilitate discussions and engaging youth participants.

A short video explaining the project was sent to teachers at the three secondary schools (one school in each of the communities visited), who showed it to their classes to help identify potential participants. In two of the schools, four classrooms with 5 to 11 students in attendance on the days of the activity agreed to participate collectively as a class. In the other school, photovoice was conducted with one group composed of students from different classrooms who were up to date on their coursework, as no teacher wanted to dedicate specific class time to the activity. Overall, 51 students (25 females and 26 males) between the ages of 12 and 18 participated. All of the youth provided written assent to participate. Five students did not return their parental consent forms (both English and Inuktitut versions were sent home by the teachers). These students were not excluded from participating in the photovoice activity since it was conducted during regular class time. However, their photos and comments were not included in the final data analysis.

In each school, the photovoice activities took place over 1 week between January and February 2020. Activities were facilitated by a member of the research team (MP) and a member of the health promotion team from the NRBHSS. Both had prior experience working with Inuit youth—one in a research role and the other as a teacher in Nunavik—experiences that were invaluable in preparing for the project. Nonetheless, their social positions as non-Inuit and community outsiders were recognized as potential biases and influences on the project process and were explored throughout by practising constant reflexivity (Nicholls, 2009). This included searching for meaning and understanding that may be different from their own, probing for further explanation in participants’ answers, and paraphrasing answers back to participants to check understanding, as well as having daily check-ins to reflect on the activities of the day. Having a representative of the health board present for the project created a direct link between youth voices and policymakers, a key component of photovoice. The youth were frequently reminded that what they shared would be reported back to those in decision-making positions at the health board.

### Data collection and analysis

Over the course of the week, the youth participated in three 2-hour sessions. In the first session, ethics of taking photographs were discussed and the youth were briefly shown how to use the instant print film cameras. Ideas for photos that could answer the question: “What in your community makes you feel healthy?” were brainstormed. In the second session, participants toured the community to take photos, deciding among themselves where they wanted to go and guiding the facilitators. At the end of the session, photos were gathered by the researcher for use in the next session. The youth were also encouraged to send in old photos from their phones or iPods® for topics that could not be captured during the excursion. Instant print film cameras (like Polaroid®) were deliberately chosen to encourage participants to select what they considered most important to photograph, as time required for printing reduced the number of potential images the youth could capture in the field and encouraged reflection.

In the third session, photos were laid out on the floor or on a table for everyone to see. Participants took turns picking a photo that they felt was important and explaining to the group how it was connected to health. Key words and phrases associated with each photo were written on sticky notes by the participants and facilitators and served as initial coding. The youth were given stickers to vote on the concepts most important to them. Ways in which photos could be shared in the community were discussed. In the last two communities, the discussions ended by having the youth create a collective text summarizing the discussion on their dream community. For this “dream community exercise”, each youth was asked to provide a sentence that described their dream community.

Group discussions were audio recorded for transcription purposes with all but one group consenting to audio recording. For this group, detailed field notes were taken by the researcher. Numbers are used in this paper to identify youth from group discussions.

**Fig. 1:**
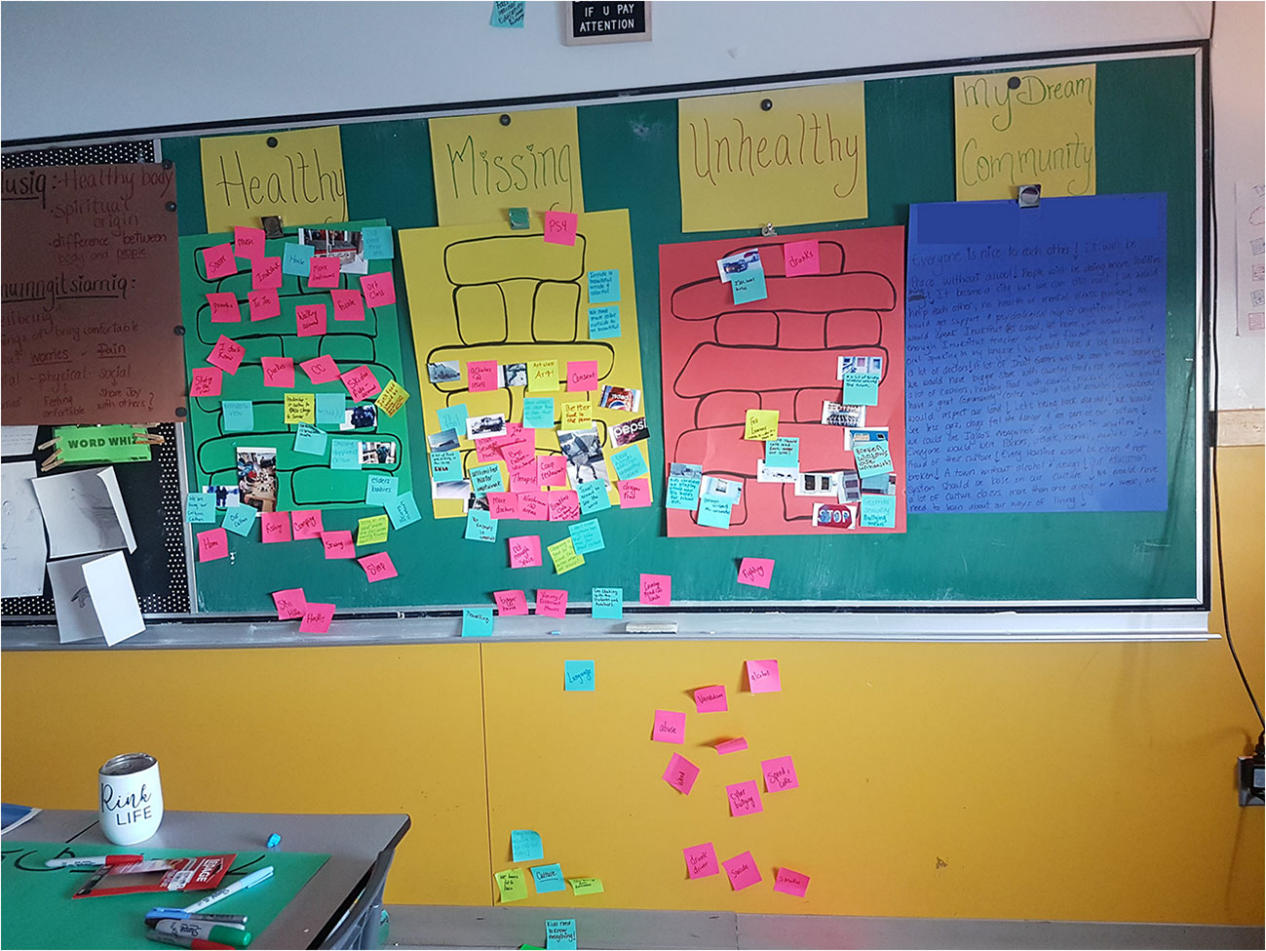
Sorting and producing initial codes for photographs on poster boards during group discussion, using an outlined image of an inuksuk as a guide

Data collection and analysis occurred iteratively and concurrently. Our interpretive process was guided by Wang and Burris’ (1997) methods for photovoice analysis, where the youth participated in selecting photos they considered most representative of their experiences; contextualizing or describing their photos orally; codifying the photos using sticky notes; and sorting the photos and sticky notes into categories and themes. After visiting the first community, the third session was adapted based on the emerging theme of the inuksuk as a representation of health—a traditional Inuit landmark, or cairn, made up of stones stacked on top of one another in the shape of a person. Instead of having the youth simply place the photo back down on the floor or table after discussing and coding it with sticky notes, the youth were asked to place their photo on an outline of an inuksuk drawn on a poster board and attached to the wall. As a group, the youth were instructed to decide where to place each photo and its associated sticky notes (Fig. 1). Using the inuksuk analogy, the bottom rocks represented concepts that were most important to health or the foundation on which all the other rocks are stacked. Two additional poster boards were added, one labelled “unhealthy” to reflect conversations raised by the youth about features of their community that were unhealthy and therefore did not belong on the healthy inuksuk, and one labelled “missing” for features that were perceived as important for youth’s health but that were absent from their community. The same three categories of poster boards were used to organize discussions in the final community.

After fieldwork was completed in the three communities, the researcher conducted thematic analysis of transcripts of group discussions and collective texts from the “dream community exercise”, as well as of an exhaustive set of photographs taken from participants’ final sorting and labelling of their photos with sticky notes. The original photos, sticky notes and posters created during the photovoice activity remained with the students at the schools. For the thematic analysis, initial codes were derived from frequently repeated words and phrases from the sticky notes. Transcripts, collective texts and photos of group sorting were read and reread in order to compile evidence for each of the codes. In this process, new potential codes were identified by the researcher. All the data were then read and reread to compile evidence for each of the newly identified codes. Once the final list of codes was established, the transcripts and photos of group sorting were then read again to help identify overarching themes among codes and refine and establish a final set of themes. Finally, the coded data extracts within each theme were re-checked to ensure internal homogeneity within themes and external heterogeneity between themes (Braun & Clarke, 2006).

Ideally, and as initially planned, the validation of the thematic analysis would have been conducted with the youth in Nunavik. But because of travel and gathering restrictions imposed by the COVID-19 pandemic, it was not possible for the research team to travel back to the communities. Because of the poor internet connection, it was not possible to directly engage in live online discussion with youth in the communities. Therefore, the validation happened remotely in fall 2020, using written comments and feedback. The research team sent teachers a handout to be printed and completed by the youth, along with a series of photos and questions related to key themes identified. The youth were asked to pick pictures they felt fit within different themes, caption photos, complete sentences, and describe and situate their importance to health with respect to other themes identified in the study. The youth were also presented with a photo of an inuksuk and asked to reflect on what they thought the photographer meant by describing the photo as healthy because “nothing is broken here”; whether things that are broken can be fixed; and how this image related to their lives. This information was then returned by email to the lead researcher. For this validation phase of the project, comments and feedback on the thematic analysis were received from six youth. Letters are used in this paper to refer to the youth who provided written feedback.

This study was approved by the Research Ethics Board of McGill University (REB File #: 169-0919). Results from this study and the initial version of the manuscript were presented, discussed and approved by the Data Management Committee of Qanuilirpitaa? survey, a committee on which sit Nunavimmiut and representatives of Nunavik organizations.

## Results

In the sections below, results are presented along the two specific objectives of the study, i.e. to identify community conditions supporting health from youth’s perspective and to explore how youth conceptualize a healthy community.

### Twelve community conditions supporting health identified by the youth

**Fig. 2:**
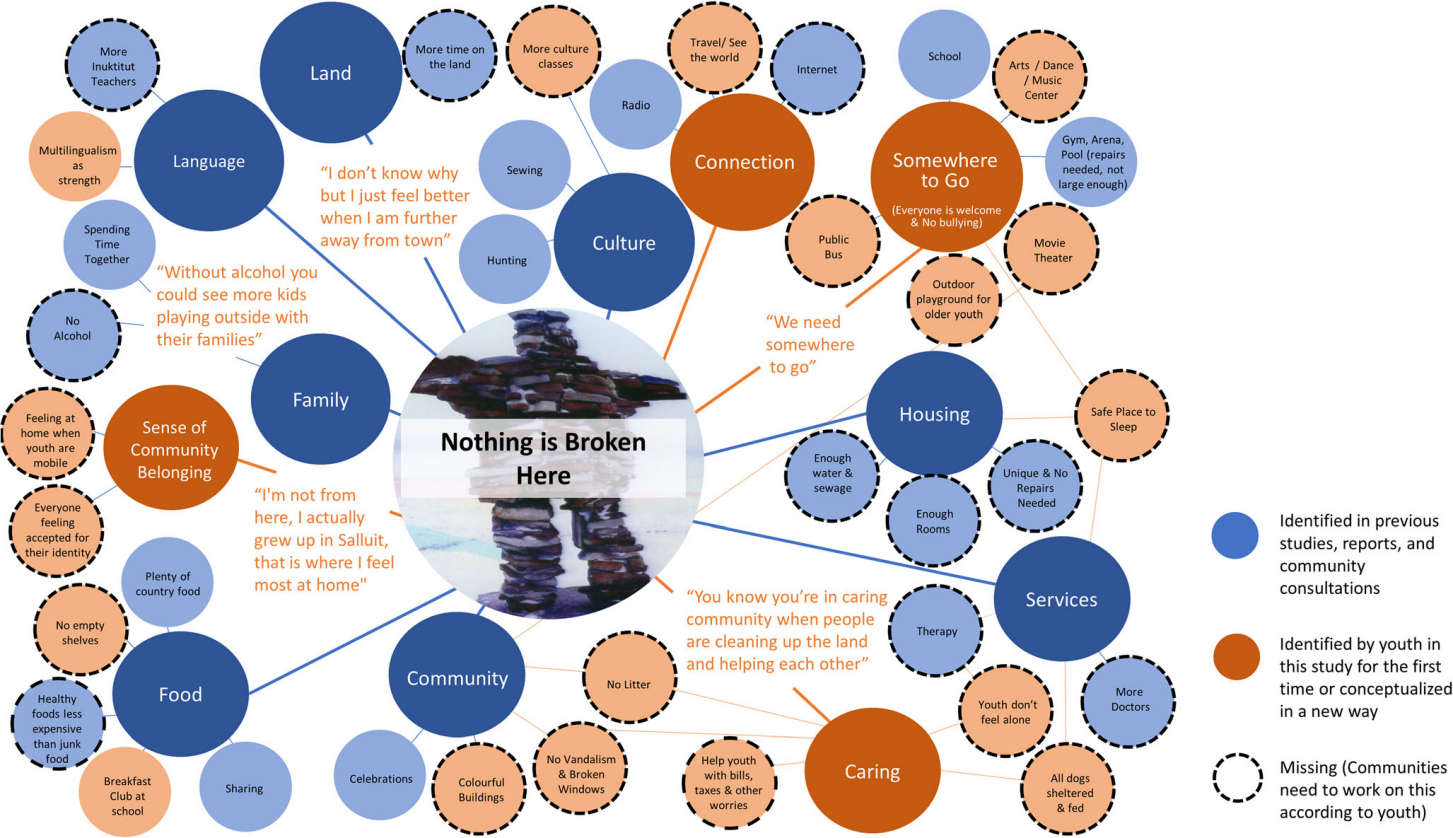
Concept map showing overview of youth understandings of a healthy community

Twelve key community conditions supporting health for youth in Nunavik were identified through thematic analysis: family, food, culture, language, identity, land, housing, services, community, connection, caring, and somewhere to go. Taken together, Table 1 and the concept map in Fig. 2 provide an overview of study results by giving examples of youth voices for each condition and illustrating how the themes identified are interrelated. The centre of the concept map represents youth’s understanding of a healthy community as a place where nothing is broken (Fig. 2). Blue circles stemming from the centre represent conditions identified elsewhere as important to Inuit health (Fletcher et al., this issue). The rust-coloured circles highlight those themes (or conditions) which were uniquely conceptualized by youth in this study. The smallest circles represent subthemes making up each condition. Some subthemes belong to more than one condition as indicated by the lines in the diagram.

**Table 1:** Summary of the 12 community conditions supporting health

Theme	Subthemes/sticky notes	Youth voices from dream community exercise
Family	Kids playing outside; playing with friends; elders and babies; alcohol-free month all the time	Parents would spend more time with their kids and their family; a town without alcohol and drugs; money will not be wasted on alcohol but on better things; without alcohol, we would have more kids playing outside; it would have more kids happy
Food	Sharing food; country food; no empty shelves at stores; breakfast club at school; more (and cheaper) fruits/berries; cheaper frozen foods; healthy foods less expensive than junk foods	We need as a community more access for food. We would have a bigger store with lots of cashiers. There would be country food like suvalik and nikkuk in the store and food that is not expensive.
Culture	Sewing; fishing; camping; hunting; traditional clothes; more culture classes	We would have a lot of culture classes, more than once a day or a week; we need to learn our ways of living. Everyone would wear parkas, atigik, kaniks and pualuks and be proud of their culture.
Language	More Inuktitut classes; multilingualism as strength	Everyone would speak Inuktitut at school and at home; we would have enough Inuktitut teachers and be fluent in writing and speaking our language.
Sense of community belonging	Everyone feels welcome	
Land	More time on the land; transport to get out on the land; dog sleds	It will become a city but we can still hunt. Let’s bring back dog sleds. We would see less gas (bad for the environment) and dogs feel the danger and are a part of our culture. We could see igloos everywhere and sleep in it anytime.
Housing	Unlimited water; more sewage trucks; no homeless; bigger houses/enough rooms; all the houses shouldn’t look the same; safe place to sleep	Every house would be clean and not broken.
Services	More doctors; more therapists; someone youth feel comfortable with	We would have a big hospital with a lot of doctors. We would get support and psychological help anytime.
Community	Celebrations; contests; community centre; movie theatre; Inuit games; colourful buildings	We would have a great community centre with a lot of activities; we could see less broken windows; a better future! Less violence; a lot of Inuit games will be seen in the community.
Connection	Transport; public bus; taxis; travel to see the world; travel between communities in Nunavik; exchanges; free trips; internet for communication and access to news and therapy	Internet is important to health because we can contact the people who are far way. Travel between communities in Nunavik is important to health because we can go see our family, friends.
Caring	No worries; memories of day care; help youth with responsibilities like bills/taxes; no trash; feed and shelter dogs; no bullying; fix what is broken	Everyone is nice to each other. It will be peace without alcohol. We would help each other, no health or mental illness problem. Everybody would respect our land.
Somewhere to go	Volleyball; hockey; running club; swimming; need larger arena; need larger gym; need a new place to hang out in; music, art and dance classes are missing; PS4; spaces should be inclusive (for boys and girls, for youth enrolled and dropped out of school) and free of bullying	We need somewhere to go. More activities would be happening for kids who do not attend school.

#### Family

In all three communities, family was considered very important to health as a source of support and comfort, with the youth expressing the desire for more quality time with family. In one community, the youth shared many positive memories of hunting and fishing with family members (and friends), showing that time on the land was an important factor in supporting family relationships. Alcohol was seen as a major challenge to family relationships and to people generally spending time together in a positive way. Youth in one community talked about how family relationships improved during an alcohol-free month campaign, and that as an outsider you could tell that the community was doing well during this month because “you could see kids playing outside with their parents”—youth 21. In this community, participants felt that family was the most important determining factor for health.

#### Food

**Fig. 3:**
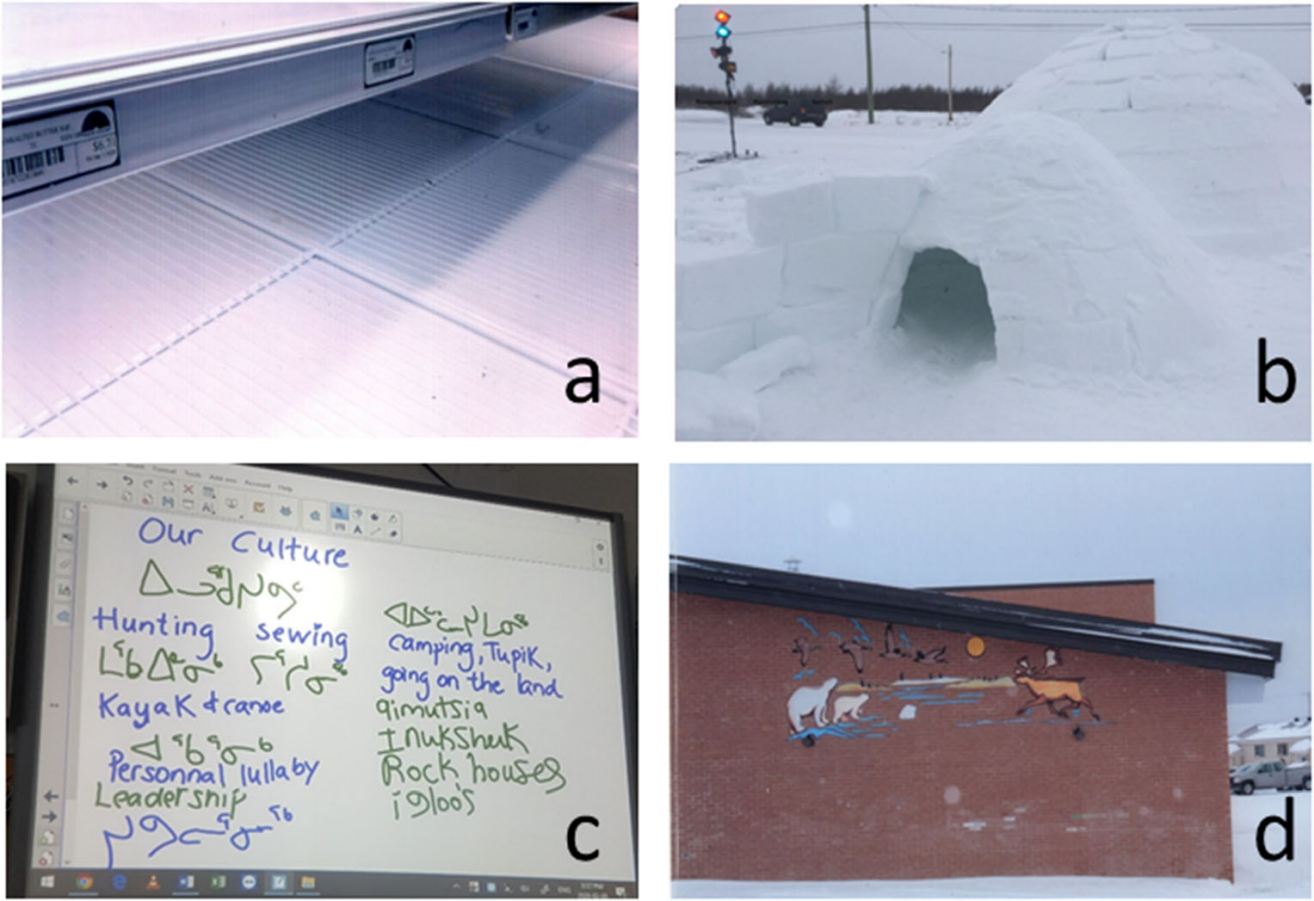
**a** Photo of empty store shelves representing the need for more access to food as a community; **b** an igloo juxtaposed with a street light and car show how in the youth’s dream community they would become a city but still be able to hunt and practise their culture; **c** during the group discussion, the youth wrote on the smart board in syllabics to emphasize the importance of the Inuktitut language; **d** the youth wished to see more of their culture reflected in their school

Access to food was an important health issue for the youth. Concerns were raised about seeing empty shelves in the store (see Fig. 3a), high prices for frozen fast foods, and junk food like pop being less expensive than healthy food. The youth enjoyed country foods such as seal, fish, geese, caribou and berries. While each community had a community freezer with free access to country foods, it became apparent that the youth were generally not aware of how stocked the community freezer was at any given time or how they could access it. In one community, the youth did not even know where the community freezer was, while others had been only once with a grandparent. The youth discussed how sharing food was healthy because “it is part of our culture”—youth 40, and explained that while country food is shared, store-bought foods (like frozen pizza) is not. School was an important source of food for the youth; they expressed a desire to see more fruits at breakfast club and to have country food at lunch. The youth were also keen to discover and try new foods and hoped to see more exotic foods at the store (for example, dragon fruit). The youth felt that money spent on alcohol could better be spent on store food and that a negative trade-off between food and alcohol was made when alcohol was purchased. In each community, the youth talked about how they would like to see a new, affordable, restaurant where they could go to eat and spend time with friends and family.

#### Culture

Culture encompassed hunting, sewing, sharing food, language, fishing, camping, storytelling, traditional clothing, sled dogs and *pisiit*—individual lullabies composed for each newborn child. While important in all communities, culture was the main topic of discussion in one of the communities. There, the youth expressed that they felt that “some villages [were] closer to their culture than [they were]”—youth 45, and that opportunities to learn about their culture were missing. In two of the communities, we heard that some form of culture or language class should be taught every day and take up more place in the school curriculum. The youth saw culture as something that could thrive alongside modernization “[in our dream community] it would be a city but we could still hunt.” This idea was represented by a photo of an igloo with a traffic light and car in the background (see Fig. 3b).

#### Language

Speaking, writing and learning Inuktitut was seen as healthy. The youth were happy to share their reflections during group discussion by writing in Inuktitut on the smart board (see Fig. 3c). There were mixed feelings of the use of English and French in the communities and having to choose between the two for attending school after grade three. For some, speaking more than one language was seen as a sign of intelligence and adaptation, but for others, it meant a loss of culture. The youth expressed challenges with learning in a second or third language and struggling with schoolwork, as well as a lack of resources to learn Inuktitut…. “[in our dream community] we would have Inuktitut teachers and be fluent in writing and speaking our language.” (from the “dream community” exercise).

#### Sense of community belonging

Sense of community belonging was a term suggested by the researcher to summarize the importance youth described of feeling at home in the community in which they were living. While taking photographs, several participants shared that the community where they were currently residing was not where they considered to feel at home, both for themselves and for many of their friends. This was usually because they grew up in another community. In one of the communities, the youth had us stop at the group home—a service which offers social, rehabilitation and integration services to youth experiencing behavioural, psychosocial or family difficulties, to see if their friends were there, only to find out they had been moved to another community. This highlights the sometimes transient nature of young people’s lives in Nunavik and speaks to the challenge of forming a sense of belonging, creating and maintaining rooted friendships. In one community which neighbours a First Nation, the participants who shared both Inuit and First Nation heritage viewed sense of community belonging as one of the most important factors for health. The participants took a photo of the First Nation high school (see Fig. 3d) as a representation of one of the many choices they feel pressured to make daily with an impact on their sense of belonging to their community. For example, having chosen to attend the Inuit school, participants felt that that they were missing out on opportunities to learn about their First Nation language and culture, knowledge that would help them feel a part of that specific cultural community. The reverse was also true; had they chosen to attend the First Nation school, participants felt that they would have been missing out on their Inuit culture. They emphasized that joint spaces connecting the two communities, such as a mixed First Nation and Inuit school, are needed.

#### Land

**Fig. 4:**
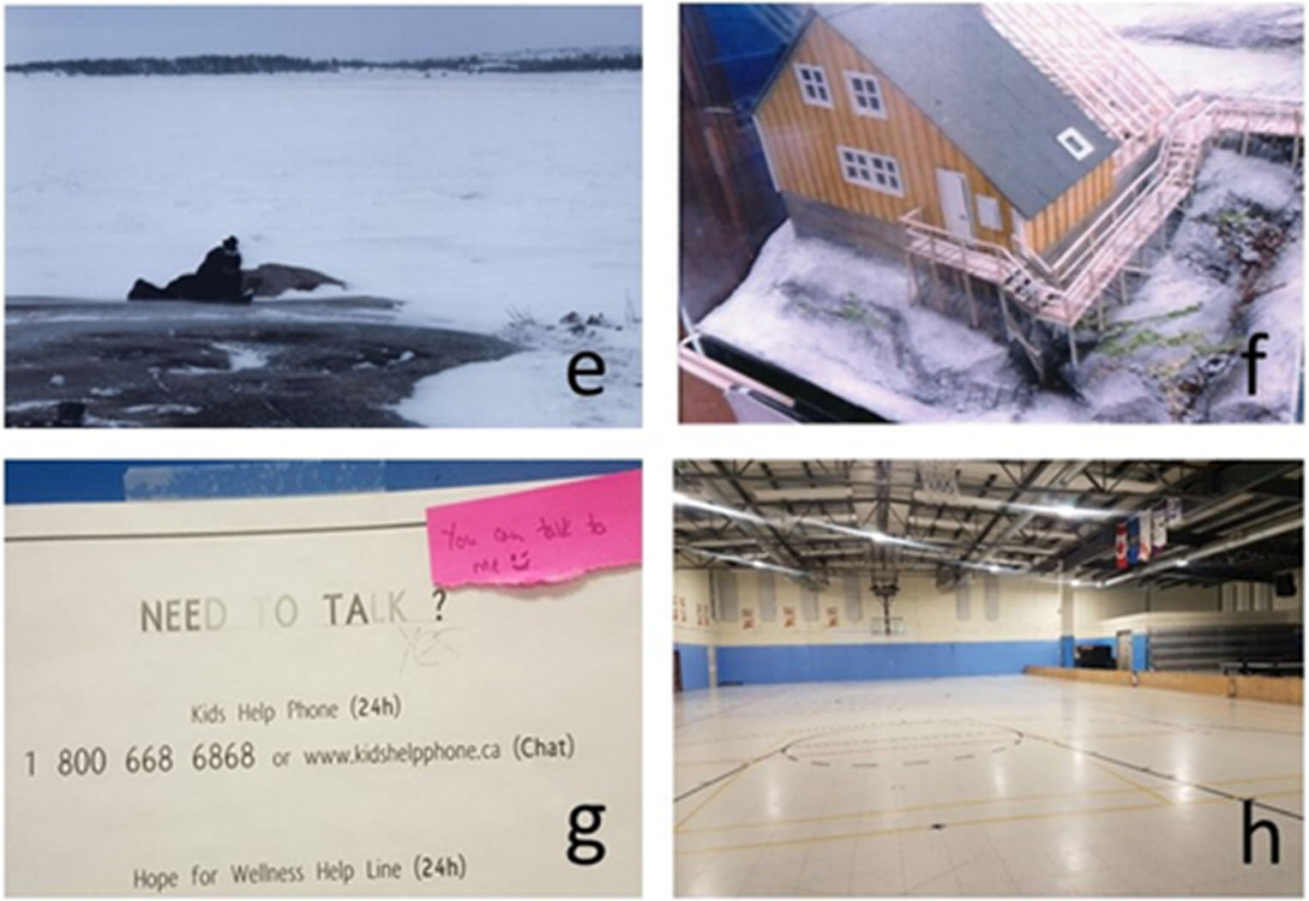
**e** The youth enjoying time out on the land taking photographs; **f** picture of a model home; this home is a bit different than most houses in the community and shows the importance of variety in building structure; **g** picture of a poster listing resources. There was consensus that the youth needed to talk more about their emotions and their lives; **h** photo of the community gym which is an important place for activities, but the youth felt was not large enough to accommodate all the activities the youth would like to see in the community

Time on the land was the most frequently mentioned theme in one of the three communities, where it was felt that youth did not spend nearly enough time outside. In this community, participants took us to three outdoor gathering spots, jumping in the snow to take photos (see Fig. 4e). While they could not explain why, the youth expressed “feeling better when they [were] further away from town”—youth 8. The youth would like to see more land-based programming and survival courses, and to spend more time on the land as part of their school curriculum. An outdoor playground or climbing wall for older youth was suggested. In one community, the youth suggested that there should be opportunities to sleep in an igloo at least once every winter. Transportation was a major barrier to getting out on the land, and the youth would like to see more accessible transportation options.

#### Housing

Housing was identified as an important component of community health, and participants were particularly concerned about homeless people in their community. The idea that everyone should have a place to stay was tied to the idea of caring for the entire community. The youth specified that housing should be new and proper and have a sufficient number of bedrooms and space for the family living there, and that there should be enough water and sewage trucks “so that [they] would never have to worry that [they would be without]”—youth 38. Not only is more housing needed, but the participants would like to see a greater variety of housing. While looking at miniature models of homes at the landholding (see Fig. 4f), one youth joked that because all the houses are the same “you always know where the bathroom is”—youth 45.

#### Services

Services referred to the need for more doctors and therapists, although the youth debated on both these points. While some of the youth felt there should never be a need to leave the community to access services calling for larger hospitals and more doctors, others welcomed the opportunity to travel outside the community for medical appointments and to access other specialized health services in the city. When it came to mental health, there was general consensus that the youth needed to talk more about their feelings and “not put things at the back of their heads”—youth C, and that there were too many suicides among young people. However, several pointed out that many youth do not feel comfortable speaking to strangers, highlighting the importance of having local people trained in supporting youth mental health, and not just more outside therapists who fly in and are considered to be strangers (see Fig. 4g).

#### Community

Community encompassed the buildings and streets as well as community-wide activities such as parties, celebrations and festivals which are centred around the community gym (see Fig. 4h) or community centre. The youth identified colourful buildings and outdoor murals as supporting health in the community as well as outdoor activities like snow sculpture competitions. The need for a movie theatre with regular movies being screened was identified as a need by the youth in all three communities.

#### Connection

**Fig. 5:**
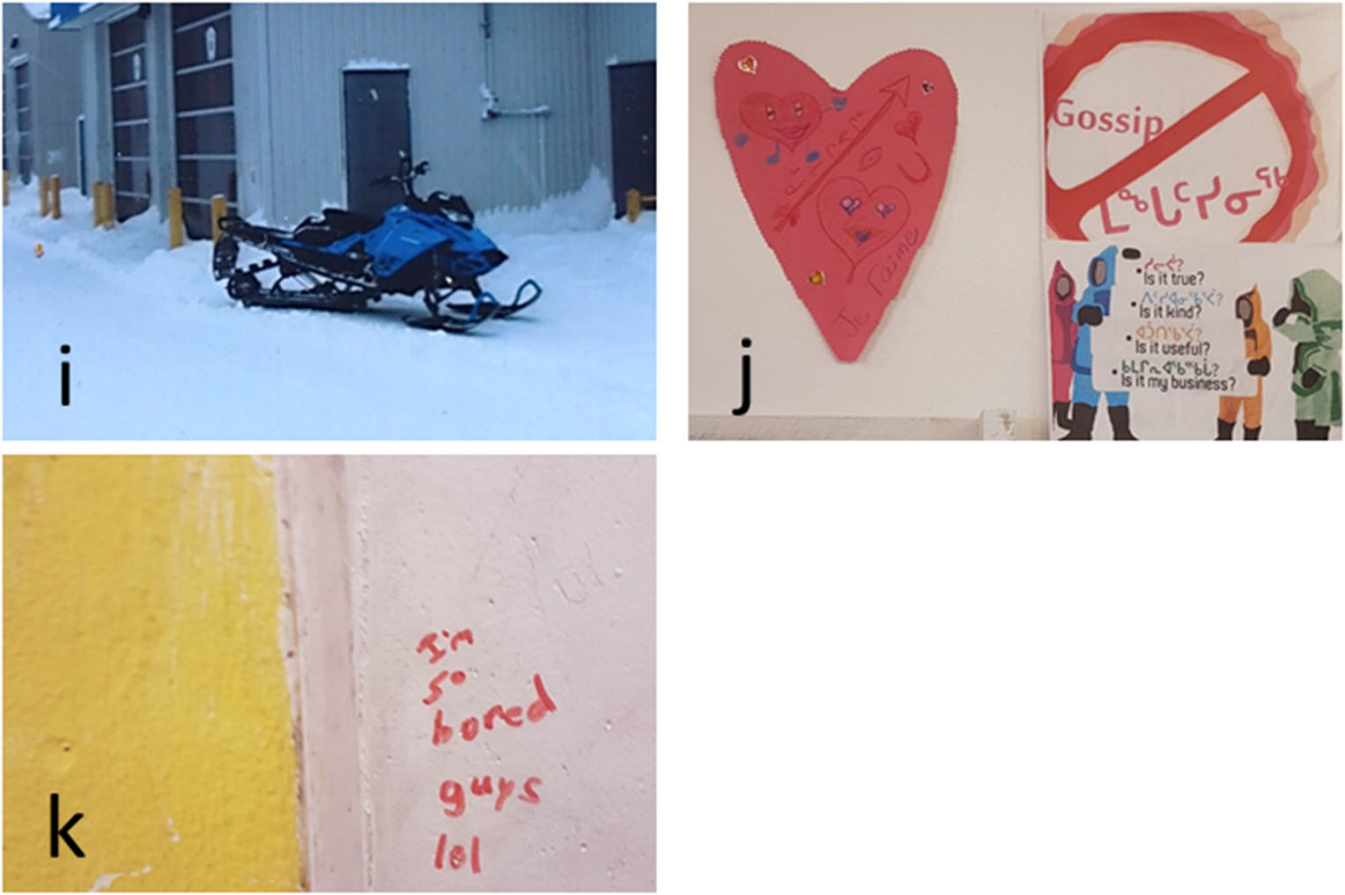
**i** A photo of a skidoo in community one highlights the need for more accessible transport for the youth to get around the community and get out on the land; **j** photo of posters representing the theme of caring; **k** writing on the wall at a youth centre representing the need for more spaces and activities for youth

Connection was a term suggested by the researcher to encompass the themes of transport, travel and internet. Transport was seen as important to help youth get to activities, particularly in light of harsh winter weather and dangerous drivers which made walking on roads unsafe. To this end, the participants would like to see a public bus that runs past 5 p.m. and more taxis, as well as more access to recreational vehicles to get out on the land. In one community, the idea of a skidoo rental shop was raised to help meet this need (see Fig. 5i). The participants equally identified a need for affordable opportunities for travel both within Nunavik, to see family and friends and meet other youth, and to communities outside of the region, to learn about different people and cultures. Internet was considered important to health in so far as it connects communities with news, friends and family in other places, and mental health resources such as online counselling. However, some participants felt that internet was unnecessary and even a negative thing in their community.

#### Caring

Caring was a broad term used by youth to discuss sheltering and feeding dogs, maintaining the land and community infrastructure (through reducing littering and vandalism), and helping every member of the community to not feel alone, particularly youth. When asked to complete the sentence “You know you’re in a caring community when…” one youth wrote “people are cleaning up the land and helping each other”—youth E. The youth described feeling alone in their responsibilities such as paying bills and providing for younger siblings. In one community, the first place the youth took us to take photos was the day care because this place was representative of a time when they did not have any worries and felt cared for—they were fed, had a big space to play and do activities, and felt a sense of accomplishment when they graduated. When asked why caring was important for health, one youth responded “because they don’t want us to feel alone”—youth C (see Fig. 5j). Another responded that caring was important for health because “it gives you motivation on anything when you know you’re cared for”—youth B.

#### Somewhere to go

The youth repeatedly raised the need to have somewhere that they could go. These included places where they could participate in cultural activities as well as sports and other physical activities, places where they could forget about their worries and have fun. Youth-designated places were equally important to give youth the opportunity “to talk about their problems and what they like and don’t like in the village”—youth D. We heard from teachers that the youth have also talked about needing spaces to find refuge from drinking or violence in the home, particularly at night. While spaces for youth such as the gym, youth centre, and arena exist in all communities, these were viewed as not being large enough to accommodate the youth population. Some of these spaces were identified as being in a state of disrepair and as lacking washrooms which made it difficult for the youth to spend time there. Another highlighted issue was the lack of consistent programming, or needing to share the space with younger children who had priority over the space at different hours of the day, meaning that the youth did not always feel like they had somewhere to go. Additionally, the participants wanted to see more activities and games at the youth centre (see Fig. 5k). Creative activities such as art, music and dance classes were highlighted in all three communities as being an unmet need, while sports were seen as more of something that existed but that programs could be expanded. Concerns around the sometimes-gendered nature of activities were raised. For example, some boys felt that they had less access to creative activities than girls did, whereas some girls felt that there were not enough sports opportunities for them as compared with for boys. When asked what the youth could do to improve spaces in their community, we heard that the youth should “clean it, rebuild it and don’t break stuff”—youth D.

### Youth conceptualized a healthy community as a place where nothing was broken

Throughout the project, the youth spoke about communities being unhealthy because there were “too many broken people”—youth 28. “Broken people” were characterized by poverty, drunkenness, engaging in vandalism, violence and suicide. It was also brought up numerous times that there was a lot of broken infrastructure in the community and that this was both unhealthy and caused by people experiencing many kinds of difficulties, trauma and loss. For example, when discussing the cause of broken windows and graffiti in one community in relation to the theme of caring, we heard that “broken people break things”—youth 42. The inuksuk was an image that resurfaced throughout the project in the youths’ photographs and discussions (Table 2). This seemed to be an overwhelmingly positive and healthy image for the youth with one describing their picture of an inuksuk as a healthy image because “nothing is broken here”—youth 39. In the validation phase, when presented with this same image and quote and asked to reflect on what they thought the photographer meant, the youth wrote about culture as something that could not be broken, although “some people forget”—youth A. No youth felt that things that were broken could never be fixed. One youth noted that an inuksuk was a representation of how things that are broken can always be fixed or put back together “because it’s made of flat rocks”—youth B. Just like a broken glass, while it may not go back together perfectly, one youth noted, “we can put it back together”—youth F. The image of an inherent potential of building anew suggests an optimism behind the difficult descriptions of “brokenness”.

**Table 2:** Reflections of youth on the image of the Inuksuk with the caption “nothing is broken here” (centre of Fig. 2)

Youth	What do you think the photographer meant when they said “nothing is broken here”?	Can things that are broken be fixed? Sometimes? Always? Never?	How does this relate to our lives?
**A**	Our culture can’t be broken but some people forget	Sometimes, because we can’t fix everything	Sometimes the sewage can be fixed
**B**	They’re brave	Always, because it’s made of flat rocks	We love it
**C**	Because it can’t be broken	Sometimes	Always
**D**	Because this never breaks	Sometimes	Problems
**E**	Nothing is mistaken	Sometimes	Everything in life
**F**	Our culture is still alive	Sometimes our emotions can be fixed	Look, if a glass breaks it can’t go back to perfect but we can put it back together

## Discussion

This study aimed to elicit understandings of community health among Inuit youth in Nunavik. Through a series of photovoice activities, 12 conditions supporting community health were identified across three communities. Together, these conditions contributed to youth representations of a healthy community as a place where “nothing is broken”.

Themes of family, food, culture, language, land, housing, services and community closely relate to descriptions of the importance of these determinants of Inuit health elsewhere, and serve to confirm existing findings (Fletcher et al., this issue; Inuit Tapiriit Kanatami, 2014). Four themes in this study stand out as unique because of the way in which they are conceptualized and prioritized by youth: connection, caring, somewhere to go, and sense of community belonging. Connection (including transport, travel and internet) highlights both the desire for youth to explore and learn about new places, as well as the day-to-day challenges in fulfilling crucial determinants of health like being on the land and visiting family, for which transportation is needed. Caring was another important condition identified, encompassing caring for people, for the environment, and for dogs in the community. The youth held a holistic view of the community where the importance of showing care extended to relationships with all living things and the environment. The youth felt healthier when they could visibly see signs of caring in their community, such as dogs being fed and buildings being maintained and not vandalized. In identifying somewhere to go as an important condition supporting community health, the participants drew attention to the fact that even if there are spaces for youth in the community, such as youth centres and sports programs, these spaces may be insufficient to accommodate the size and diverse needs of the youth population, and that a closer consideration of factors like the inclusiveness of these spaces, as well as the development of regular programming, is needed. Finally, in discussing the importance of having a sense of community belonging, the participants drew attention to the challenges youth sometimes face in feeling connected to their communities and the importance of efforts aimed at helping everyone feel welcome.

Throughout the study, the image of the inuksuk repeatedly surfaced as a representation of health. In addition to being an image chosen by youth participants, the inuksuk presents a potentially appropriate image for use in health promotion with Inuit youth for several reasons. First, because the rocks are stacked in the form of a person, this reflects the centrality of people, family and relationships in the youths’ understanding of health (as reflected in the theme of family in this study). Second, because the individual rocks themselves cannot be broken, this represents that even in times of struggle or deficits to health in the community (which can be thought of as some of the rocks having fallen or gone missing), an inuksuk can ultimately be rebuilt. This aligns with the youths’ expressed belief in this study that even things that are broken can be fixed as well as with youths’ perception of their own role in creating a healthy community—rebuilding what is broken.

Several scholars have argued that recognizing hurt in the past is important to moving forward in Indigenous communities (Joo-Castro & Emerson, 2021; Nicolai & Saus, 2013). We heard in this study that “youth feel cared for when a person does understand our pain and our past”—youth F. While others have argued the importance of moving away from conversations of “brokenness” in order to focus on a strengths-based approach (Wood et al., 2018), this study showed that even in taking a strengths-based lens, conversations of community health with youth could not be separated from discussions of brokenness as this shaped youth’s realities. This is an important finding for shaping effective health promotion work in this population, particularly if it can be reframed in a positive way as the youth did in this study through shifting the conversation to rebuilding. A focus on rebuilding simultaneously recognizes hurt in the past while looking forward to the future. This analogy aligns with the framing of existing community-based initiatives in Nunavik which emphasize the importance of unlearning trauma for youth well-being (Nunavik Regional Board of Health and Social Services, n.d.), as well as initiatives in other parts of the country, like the “Build Them Up” campaign which works on improving mental health for youth in Alberta through a focus on building resilience (Alberta Children’s Hospital Foundation, 2020).

While all 12 conditions supporting community health were discussed by the youth from all communities, in each community, a different condition (land, family and culture) emerged to dominate the photographs and discussions. Such community differences highlight how specific and local contexts shape the health priorities of the youth. This finding has implications for tailoring existing services and infrastructure and provides caution against a one-size-fits-all approach to health promotion. In having youth collaboratively decide what was most important for community health, or what belonged at the base of the inuksuk, this study provides a starting point for youth to address health issues in their community by breaking down a large concept like health into manageable chunks and identifying group priorities. This interactive discussion method may be useful in facilitating discussions with other youth in Inuit communities.

Over the course of the project, we faced several challenges that had an impact on study design and in conducting the study. Lengthy ethical approval processes combined with funding deadlines meant that relationship-building with the youth prior to arrival was difficult and made it unfeasible to engage one youth from each community as a research assistant as originally planned. Instead, we decided to involve all youth in validating and providing feedback on results. Yet this too was made difficult due to the impacts of the COVID-19 pandemic, including a travel ban to the region for outsiders and the abrupt ending of the school semester shortly after fieldwork was conducted. Even in the fall of 2020 when school resumed, following up with teachers was difficult. We had also planned activities for the youth to share photos in their communities; these were indefinitely paused because of COVID-19 restrictions. One limitation of this study was that fieldwork was limited to 1 week in each community. While the 1-week time frame was chosen in a conscious effort to minimize time taken away from course work for students, this limited the possibility of exploring any one topic in greater depth. Future research projects may choose to look more closely at the individual conditions identified in this study.

Photovoice was seen as a fun and effective way of engaging youth. We heard from teachers that while the day-to-day conversation can be quite negative, they were surprised by the overwhelmingly positive answers that this project elicited from participants, suggesting that this is an effective method for framing health in a strengths-based perspective. Methodologically, we noted that the richest discussions often happened outside the school, while we were taking photographs. However, these discussions were not recorded. Future research would benefit from a “go-along” approach (Carpiano, 2009) where outdoor activities and photo sessions are recorded. Additionally, several gender-based differences were raised by participants suggesting that separating participants by gender in future studies and exploring similarities and differences may yield interesting results.

## Conclusion

This study provides regional and culturally specific insight into community health understandings of youth in Nunavik. In doing so, it responds to community-based calls for research to inform health interventions targeted at this population and contributes to efforts for improving community health more generally.

## Contributions to knowledge

What does this study add to existing knowledge?

- This study serves to confirm existing findings on the factors that are important for Inuit health, while raising fresh perspectives that are relevant to the younger generation.
- Despite being a large and growing segment of the Inuit population, youth are rarely asked their perspectives and opinions. In soliciting the opinions of the youth, this study responds to an important generational research gap, providing a more complete picture of the community conditions that support Inuit health.

What are the key implications for public health interventions, practice or policy?

- Findings from this study contribute to identifying pathways to improve youth health in these communities. Towards this end, the image of the inuksuk might prove to be a powerful tool for discussing and engaging youth in conversations around community health because of the way in which it symbolizes how they conceptualize health as “nothing is broken”.
